# miRNA as a Biomarker for the Early Detection of Colorectal Cancer

**DOI:** 10.3390/genes15030338

**Published:** 2024-03-05

**Authors:** David Coleman, Scott Kuwada

**Affiliations:** 1 John A. Burns School of Medicine, University of Hawaii, 651 Ilalo Street, Honolulu, HI 96813, USA; 2 University of Hawaii Cancer Center, 01 Ilalo Street, Honolulu, HI 96813, USA

**Keywords:** colorectal cancer, CRC, miRNA, microRNA, biomarker, CRC screening

## Abstract

MicroRNAs (miRNAs) are short, non-coding RNA segments that can be detected in a variety of clinical samples, including serum, stool, and urine. While miRNAs were initially known for their effect on post-translational gene expression, the last decade of research has shown them to be promising biomarkers for the detection of many types of cancer. This paper explores the use of miRNA detection as a tool for colorectal cancer (CRC) screening. We discuss the current state of miRNA detection, compare it to the existing CRC screening tools, and highlight the advantages and drawbacks of this approach from a clinical and logistical perspective. Our research finds that miRNA-based tests for CRC show great potential, but that widespread clinical adoption will be conditional on future research overcoming key hurdles.

## 1. Introduction

Screening tools play a crucial role in the early detection and prevention of CRC. These diagnostic methods have evolved over the years, and now include a variety of established techniques, as shown in Table 1.

Among the current CRC screening tools, multi-target stool DNA (mt-sDNA) tests, fecal immunochemical tests (FIT), and fecal occult blood tests (FOBT) are non-invasive methods that detect blood or CRC-specific biomarkers in the stool, potentially indicating the presence of cancerous or precancerous lesions [6]. Stool-based CRC screening tests are hampered by relatively lower sensitivity and specificity compared with colonoscopy, plus the need for the patient to retrieve fecal samples. FIT-based programs, which are among the most common worldwide, are limited by a low sensitivity for detecting patients with advanced adenomas [7]. Furthermore, current guidelines dictate that a positive result for these stool tests should be followed by colonoscopy, as the implication of a positive result is the possibility of underlying colorectal neoplasia [6].

Blood tests for CRC screening have been developed as an alternative to stool tests, and there is broad acceptance of routine blood testing in healthcare. Carcinoembryonic antigen (CEA), a biomarker for CRC, has been studied for CRC surveillance. However, this is only effective when the initially diagnosed cancer was positive for CEA, which is the case for only approximately 70% of colorectal cancers [8]. Another non-invasive biomarker that can be tracked for the surveillance of CRC is carbohydrate antigen 19-9 (CA19-9), but it is of limited clinical use due to its low sensitivity and specificity [9]. Septin9 has also been tested for CRC screening, but it has been shown to perform significantly better for advanced stage CRC (III–IV), making it a suboptimal tool for the screening and early detection of CRC [10]. One of the problems facing blood-based testing is the potential for elevated circulating biomarkers due to health conditions unrelated to CRC, resulting in concerns regarding the diagnostic challenges created by false positive results.

More recently, microRNA (miRNA) has been studied as a CRC screening modality because many miRNA sequences are aberrantly expressed by colorectal neoplasms [11]. miRNAs are stable, short, double stranded RNA sequences that can be detected and quantified in clinical samples through PCR analysis. The molecular stability of miRNA supports the scalability of this modality, which is a prerequisite for widespread clinical use [12]. miRNAs have also undergone extensive testing as stool-based CRC tests because they are directly expressed by colorectal cancers and neoplastic colon polyps [13].

The current recommendation for all of these non-invasive tests is a colonoscopy in the case of a positive or clinically significant (CT colonography) result [6]. Colonoscopy is unique in that it allows for the removal of precancerous polyps, the precursor lesions of colorectal cancer, during the screening process itself. Since the detection and resection of precancerous polyps has been clearly shown to reduce CRC, the ultimate success of all non-invasive CRC tests relies on the colonic or surgical resection of colorectal neoplasms. However, the downsides of colonoscopy are the associated cost, the invasive nature of the procedure, the requirement of bowel preparation, and the risk of serious complications [14]. There have been other direct visualization CRC screening methods, including flexible sigmoidoscopy and CT colonography, but the current usage of these modalities for CRC screening is low. Flexible sigmoidoscopy, although effective at reducing CRC mortality, is inherently limited by the incomplete visualization of the colon [14]. CT colonography requires bowel preparation, additional tests for clinically insignificant extracolonic findings, and a subsequent colonoscopy if lesions, including benign polyps, are detected.

While colonoscopy is considered the gold standard against which other CRC screening tests are compared, it cannot be universally applied to screening eligible populations due to the reasons mentioned above. Risk stratification approaches have been adopted to help bridge that gap, but the research into this topic has struggled to demonstrate clinical effectiveness and cost effectiveness in comparison to the current practice [8]. An important risk factor for CRC is age, and the risk of precancerous polyps clearly increases with age [15]. Taking this into account, it would seem to make more sense for older individuals with a close family history of CRC to undergo colonoscopy instead of stool-based tests due to these individuals’ higher pre-test probability for colorectal polyps, which could trigger positive stool-based results and thus generate costs for two CRC tests rather than one. For younger screening-eligible individuals with average risk for CRC, however, perhaps a better option would be a non-endoscopic test that is specific and sensitive for CRC, non-invasive, cost-effective, and generally acceptable to patients. Unfortunately, this optimal tool for CRC screening arguably does not yet exist.

Several comprehensive reviews of miRNA-based CRC and advanced adenoma detection studies have been published in the last few years [10,13,16,17,18,19]. These large reviews demonstrate the ample number of studies proposing aberrantly expressed miRNA species as potential CRC screening biomarkers, raising the question of whether or not miRNA-based tests are ready for clinical adoption in CRC screening. For this review, a literature search was conducted primarily through the databases of PubMed, Science Direct, Web of Science, and Embase. We acknowledge the high quality and comprehensive reviews mentioned above, and instead focus on the timing of the implementation of miRNA in CRC screening. Thus, our literature search included a number of studies that investigated the utility of specific miRNAs in validation cohorts as biomarkers for CRC. Analysis of these targeted studies helps us understand the accuracy and strengths of miRNA-based tests and identify any potential shortcomings in the developing science around miRNA-based CRC screening. Many of the previously cited studies have discussed current and potential challenges in implementing miRNA in CRC screening, and we expand the discussion around key issues.

## 2. miRNA-Based Detection of Colorectal Cancer

miRNAs are small RNA strands that can be detected in blood, stool, or urine samples, meaning that their collection and use in CRC screening or surveillance is less invasive than colonoscopy. Many recent studies have observed altered expression of specific miRNAs in the early stages of colorectal carcinogenesis, providing a potential avenue for the prevention and early detection of CRC [20,21,22,23,24,25]. miRNA can be exported from cells through vesicular trafficking or can be shed in cell fragments during apoptosis [26]. The discovery of candidate miRNAs typically originates from differential expression assays of miRNAs between human colorectal cancer and normal colon tissue specimens. The resulting differentially expressed miRNAs are then further selected by testing their performance in blood, stool, or urine samples from the same case and control patients. Some studies cited then go on to test the candidate miRNAs in previously untested cohorts. While some research has focused on individual miRNAs [21,23,27], other studies have used panels of miRNAs that, when analyzed as a group, detect the presence of CRC with a sensitivity and specificity equal to or greater than traditional non-invasive screening methods [20,22,28,29].

While there are currently no federally approved miRNA tests for screening colorectal cancer, a serum miRNA panel for detecting gastric cancer was approved by Singapore’s Health Sciences Authority in late 2019 [30]. This panel, marketed under the name GASTROClear^®^, uses 12 miRNAs to screen for gastric cancer with a reported sensitivity of 87.0%, a specificity of 68.4%, and an AUC of 0.92 in their validation cohort [31]. Although the targeted miRNAs are different in gastric cancer, Singapore’s approval of a miRNA-based cancer screening test is a good sign for the future adoption of similar tests for CRC.

Many individual miRNAs have been studied in the context of CRC screening, and several detailed reviews have been conducted on this topic [10,13,16,17,18]. Among those reviews, certain miRNAs have emerged as particularly strong candidates for further study (Table 2). This list is not exhaustive, and there are several limitations of showcasing miRNAs like this. First, all of these miRNAs have been researched in multiple studies using non-standardized techniques. Naturally, the exact performance of these miRNAs in CRC screening varies across these studies, influenced by factors including methodological and test population differences. The numbers included in the table below represent the reported mean (or sole) values from Santos et al. [16], and sensitivities and specificities from other studies are not shown so as to minimize the risk of misinterpretation. The rightmost column includes citations to many of the original studies from which these numbers were derived. Second, this table highlights individual miRNAs; however, many of them have also been analyzed as part of one or more panels composed of multiple miRNAs. It is worth noting that recent studies have shown that miRNA panels generally provide greater sensitivity and specificity for CRC than individual miRNAs [13]. The numbers reported below represent their performance individually and not as part of a panel. Despite these limitations, we thought it would be beneficial to call attention to some of the most promising candidate miRNAs that may later be included in clinical CRC screening tests.

The utility of miRNA for CRC detection was first studied in blood. A good example of this is a case–control study performed by Liu et al. [40]. These researchers purified miRNA from plasma samples, evaluating the predictive power of ten different miRNA sequences for CRC risk. Three of these—miR-29a (sensitivity = 0.961, specificity = 0.316), miR-125b (sensitivity = 0.932, specificity = 0.326), and miR-145 (sensitivity = 0.854, specificity = 0.438)—were significantly associated with CRC. Using ROC curve analysis to assess the predictive value of these miRNAs, the inclusion of the miRNAs into the model increased the AUC from 0.61 to 0.71, showing that these miRNAs can be used to enhance predictive models for CRC.

Instead of analyzing miRNA in blood samples, some researchers are investigating the use of urine samples, obviating a needle stick, for miRNA-based CRC screening. One case–control study, published in 2022 by Iwasaki et al. [25], used a two-miRNA panel consisting of miR-129-1-3p and miR-566 to distinguish CRC from healthy control patients. The elevated expression of these miRNAs in patients had good predictive power in their training cohort (AUC = 0.811, sensitivity = 0.807, specificity = 0.707), which was supported by their validation cohort (AUC = 0.868, sensitivity = 0.889, specificity = 0.762). Although urine contains less miRNA than the more commonly studied serum/plasma and stool samples [25], it is easier to collect than stool or blood, and the miRNA within it is stable under clinical storage conditions [45]. That being said, the vast majority of research into using miRNA to screen for colorectal neoplasms is focused on blood and stool samples, and this is unlikely to change in the near future.

In general, the use of body-fluid-based CRC biomarkers may be hampered by the lack of specificity of the markers for CRC, and the investigation of false positive test results may therefore be complicated and expensive. In stool samples, however, the ability to detect miRNA directly expressed by colorectal tumors may yield increased specificity for CRC because some of the fecal miRNAs originate directly from the CRC. Pardini et al. [20] used a machine learning model to assess miRNA profiles in stool samples of patients from two European cohorts with a combined sample size of 479. Utilizing machine learning to discern the miRNAs that are most highly associated with CRC allows for a much broader survey of the myriad of aberrantly expressed miRNAs in many CRCs. Their goal was to identify a fecal miRNA signature that could distinguish CRC patients from controls, and they found 25 miRNAs that were differentially expressed in CRC patients. From that subset, they isolated a panel of five miRNAs, consisting of miR-607-5p, miR-6777-5p, miR-4488, miR-149-3p, and miR-1246, that offered the highest discriminative power, with the AUC of models incorporating this panel ranging from 0.86 ± 0.01 to 0.96 ± 0.01. This study was unique in the manner it used to identify miRNAs of interest, with their machine learning-based approach identifying many miRNAs that had not previously been closely studied with respect to CRC. They also compared their stool sample results to a subset of plasma samples from their study population, and found evidence that stool samples may offer greater sensitivity than plasma samples for the detection of CRC.

Notably, there is not yet a clear consensus on the ideal source for miRNA collection, and comparative studies are needed. While miRNA can be found and analyzed in blood, stool, or urine, there are theoretical and practical advantages and disadvantages to each of these approaches. Drawing blood is convenient and low-cost, but in the case of miRNA can suffer from a lack of specificity if there is overlapping aberrant expression of certain miRNAs among cancers originating from different tissues [46]. Stool samples are likely to contain miRNA that is more specific to CRC and the gastrointestinal tract, but patient aversion to stool collection may decrease widespread adoption. Urine is easy to collect, but it contains relatively lower levels of miRNA compared to stool or blood samples [25], which may limit its effectiveness. The combination of fecal DNA and occult blood testing has emerged as a potentially enhanced approach for achieving higher sensitivity and specificity for advanced colonic neoplasms, similar to the strategy employed in Cologuard^®^ (Exact Sciences Corp., Madison, WI, USA), although at the expense of more false positive results [47,48]. We expect future research will shed light on which approach is best overall.

Another important trait of miRNA-based tools relates to how specific miRNAs are expressed in greater amounts during certain stages of CRC. By tracking this variable miRNA expression, it is possible to monitor a patient’s response to treatment or the progression of disease through purely non-invasive means. For example, a 2013 study found significant overexpression of twelve miRNAs and underexpression of eight miRNAs in patients with CRC [11]. Importantly, the levels of over- and underexpression were more pronounced in later TNM stages, showing that miRNA has the potential to track disease progression, enabling its use as a marker for prognosis.

The early detection of CRC has been a key part of the strategy for decreasing CRC mortality, since cancer survival is highly dependent on the stage at diagnosis. This concept was further studied by Shiosaki et al., who examined serum levels of CRC-associated miRNA across CRC stages compared with those of controls (no CRC) [24]. This study found that the ratio of non-vesicular to vesicular levels of miR-21, miR-29a, and miR-92a were significantly higher in early-stage CRC (I-II) compared to those ratios in the control group. The increased presence of CRC-related miRNAs in the non-vesicular fraction of serum suggests that miRNA may be transported out of or released from vesicles over time, thus leading to accumulation in the non-vesicular fraction of blood. While many studies have examined miRNA levels in cell-derived vesicles in serum or plasma, closer investigation of the ratios of non-vesicular to vesicular miRNA levels may increase sensitivity for early-stage CRC.

While miRNA-based screening has numerous potential upsides, there are several drawbacks worth mentioning. Perhaps the most significant of these is that miRNA expression can vary across ethnic groups. One study that looked into this dynamic was Bovell et al., published in 2013 [49]. These researchers investigated the expression of five miRNAs (miR-20a, miR-21, miR-106a, miR-181b, and miR-203) in tissue samples from 142 black and 239 white CRC patients. They found significant differences in miRNA expression across these groups, logically corresponding to variability in miRNA-based screening test accuracy. Since that paper’s publication, a multitude of studies have proposed a wide variety of miRNA signatures for use in CRC screening, but very few of them have acknowledged the reality of ethnic variation in miRNA expression. With miRNA-based CRC screening advancing towards adoption into clinical practice, it is important that future studies account for this variability. Doing so could allow for generalizability, while failing to do so could introduce significant ethnic discrepancy in the validity of miRNA-based assays.

As mentioned earlier, an additional concern for miRNA-based CRC screening is that of organ specificity. As an example, miR-21 is aberrantly expressed in prostate cancer, Hodgkin’s lymphoma, hepatocellular carcinoma, as well as CRC [46]. A positive miR-21 blood or stool test may be the result of many potential neoplasms, thus limiting its utility in screening for a specific type of cancer. The more unique aberrantly expressed miRNAs are to cancers of a particular organ, the more focused and thus cost-effective the subsequent diagnostic evaluation would be for a suspected neoplasm. In addition, miRNA expression profiles can vary across individual neoplasms from the same tissue of origin, adding to the challenge of implementation.

Differential miRNA expression is not unique to colorectal cancer, and many miRNAs are being studied for their utility as biomarkers for other diseases, including, but not limited to, cancer [50]. A relatively common approach to investigating miRNAs for CRC screening has thus far been to compare cases and controls, but this approach inadequately addresses the need for accurate CRC screening in complex patients who may have any number of other health conditions that can modulate miRNA expression. In their 2021 systematic review of the diagnostic performance of fecal miRNA CRC screening studies [13], Zhao et al. reported that, out of the 20 studies included in their extensive review of human trials, only 1 recruited its participants from a true screening program, thus accounting for patient heterogeneity with regard to comorbidities. The remaining 19 recruited their study participants from patients who had already been diagnosed with colorectal neoplasia.

There are multiple technical challenges that must be addressed in the research as a part of the clinical utility of miRNA detection in CRC screening. In the discovery phase, miRNA expression patterns were compared across relatively small numbers of CRC cases and healthy controls. Although some studies tested the differentially expressed miRNAs in validation cohorts, the studies were generally small and population-based. Additionally, there is currently a lack of standardization in the processes and platforms used to quantify miRNA in clinical samples. There are many methods that can be used to measure miRNA expression, including real-time quantitative reverse transcription PCR (qRT-PCR), droplet digital PCR (ddPCR), and microarray hybridization [51,52]. These methods often rely on normalization to quantify miRNA expression; however, the constancy of the expression of reference miRNAs has been called into question [53,54,55]. At the very least, this methodology varies across the research seeking to identify miRNA biomarkers for CRC, making it difficult to compare the results from different studies. Ideally, this problem would be addressed by well-designed research studies on normalization that is reproducible in large and ethnically diverse populations.

## 3. miRNA-Based Detection of Colorectal Cancer Precursor Lesions

Recognition of miRNA’s potential as a biomarker for CRC started in the late 2000s. The earliest published papers on this topic focus nearly exclusively on the detection of CRC, with no attention paid to the detection of CRC precursors. The adenoma–carcinoma sequence is well known [56], and the strength of colonoscopy in decreasing CRC incidence is based on the removal of dysplastic precursor polyps [57]. Thus, increasing emphasis has been placed on these tests’ ability to detect the precancerous polyps that develop into CRC, namely advanced adenomas and sessile serrated polyps [20,23,38,58,59,60]. An interesting example of this is a study that took the approach of assessing the feasibility of using miRNAs as biomarkers for CRC in fecal occult blood [61]. They used a hydrolysis probe-based miRNA assay to detect the miRNA in stool samples from a patient sample composed of 29 CRC cases, 31 with advanced adenomas (>1 cm diameter, villous architecture, or high-grade dysplasia), and 115 controls with normal colonoscopy. When analyzing the ability of their miRNA panel (miR-200b-3p, miR-144-5p, and miR-144-451a) to detect advanced adenomas, they ultimately concluded that it was poor, with an AUC of 0.58. However, they did discover that their miRNA panel was significantly better at detecting adenomas in the distal colon. Other studies have looked at the efficacy of miRNA-based detection of precancerous lesions for CRC, and they show more promising results (Table 3).

## 4. Discussion

Compared to other modalities of CRC screening and surveillance, there are multiple advantages to the use of miRNA. From the patient’s perspective, perhaps the most significant of these is the less invasive nature of miRNA-based testing, requiring only blood, urine, or stool samples. This contrasts with the more invasive screening methods, such as colonoscopy, that may deter individuals from undergoing regular screening [64]. It should be noted that colonoscopy should still be used in individuals at significantly increased risk for colorectal neoplasia based on pre-existing conditions. These may include prior CRC, polyposis syndromes, a history of dysplastic colorectal polyps, long-standing chronic inflammatory colitis (ulcerative colitis, Crohn colitis), a strong family history of CRC, certain hereditary cancer syndromes, and primary sclerosing cholangitis. However, non-invasive methods are known to enhance patient compliance [65], and would contribute to the feasibility of population-wide screening programs.

One of the most clinically relevant advantages is miRNA’s potential for early detection of precancerous advanced adenomas and sessile serrated polyps. Detection of neoplastic polyps before they transform into carcinoma is a significant strength of screening colonoscopy when compared to traditional non-invasive CRC screening such as FIT or FOBT [7,66,67]. However, the inclusion of advanced adenoma detection in miRNA-based screening studies has shown that miRNA may be able to detect advanced adenomas with greater sensitivity and specificity than FIT or FOBT (see Table 3). The early detection and resection of precancerous polyps has been shown to decrease both the incidence and mortality of the disease [68], and improving our ability to detect early stages of CRC directly supports this. This is one area where miRNA may have a significant advantage over other stool-based CRC assays.

Aberrant miRNA expression has been demonstrated in the stool, blood, and urine of patients with CRC, but tissue specificity will be especially important in the implementation for CRC screening. One potential avenue for addressing this is the modality of the test itself. Santos et al. [16] pointed out that the proximity of stool samples to potentially neoplastic colon cells is a point in favor of fecal miRNA testing, concluding that future studies should pay greater attention to the unique advantages of fecal miRNA. The contamination of blood samples with miRNAs that have been released from hemolysis or non-cancer circulating cells (e.g., platelets or leukocytes) [69] may again make fecal miRNA detection more specific to colorectal neoplasms in the absence of intestinal inflammation.

While much of the focus on the implementation of miRNAs in cancer screening has been on which miRNAs offer the best sensitivity and specificity for neoplasms, more attention will need to be devoted to standardization of the miRNA assays used and ethnic variation. For widespread adoption of miRNA in CRC screening, the assay used to detect and quantify miRNA species in any media will need to be reproducible and scalable, and in a cost-efficient manner. In addition, the studies cited show ethnic differences in miRNA expression, and this will require further study as world migration continues in many countries.

This review is intended to (1) describe the current state of miRNA-based CRC screening, (2) give examples of the different strengths and weaknesses of the field, (3) discuss these strengths and weaknesses, and (4) identify the hurdles that must be overcome before widespread clinical adoption can take place. In doing so, we hope to give the reader an accurate understanding of how miRNA compares to the existing CRC screening tools as well as a sense of how miRNA-based testing can complement those tools if or when it is adopted into clinical practice. However, we acknowledge that this review has certain limitations, which we describe here. The nature of novel technology is such that advancements occur constantly, meaning that published research on the subject may quickly become out of date. This review is not immune to this, and newly released literature or commercial products may soon contradict points made in this paper. There are also subjects related to miRNA and CRC that were not discussed, including the implications of cancer metastasis, disease prognosis with respect to certain miRNAs, the specifics of miRNA detection technology and recent advancements in this field, and detailed exploration of existing CRC screening tools. These subjects were omitted in the pursuit of concision and deeper discussion on the nuances of miRNA-based screening, but they merit further consideration in future research.

## 5. Conclusions

Ultimately, miRNA testing represents a promising advancement in the realm of CRC screening and surveillance. Combinatorial assays, such as fecal immunochemical hemoglobin testing and miRNA assays, may enable improved sensitivity and specificity in CRC screening. miRNA as a molecular species is stable, relatively small, and present in feces and blood, making it more accessible and less invasive than colonoscopy, the gold standard. However, future research must address the inherent variability of target miRNA expression in diverse populations, as well as ensuring reproducible results through the adoption of standardized technology. Only then can the development of miRNA CRC screening assays achieve economic feasibility and scalability. To date, there have been no sufficiently powered and large population-based randomized prospective trials on miRNA versus colonoscopy, as the gold standard, for CRC screening. There are still several issues to resolve before investing in large prospective trials. Ongoing research and robust validation are key to unlocking the full potential of this new tool, and we look forward to the insights that future research will bring in the coming years.

## Figures and Tables

**Table 1:** Established screening modalities and tests for colorectal cancer.

Screening Test	Modality	Sensitivity for CRC	Specificity for CRC
Colonoscopy	Direct Visualization	18–100% [1]	- *
CT Colonography	Direct Visualization	88.8% [2]	75.4% [2]
Flexible Sigmoidoscopy	Direct Visualization	40.74% [3]	93.33% [3]
mt-sDNA	Stool-based test	92.3% [4]	86.6% [4]
FIT	Stool-based test	73.8% [4]	94.9% [4]
FOBT	Stool-based test	50% [5]	77.87% [5]

* The specificity of colonoscopy for CRC is left blank because colonoscopy is considered the standard of reference for determining the presence of CRC. Abbreviations: CRC: colorectal cancer; CT colonography: computed tomography colonography; mt-sDNA: multi-target stool DNA; FIT: fecal immunochemical test; FOBT: fecal occult blood test.

**Table 2:** A selection of promising miRNAs for use in colorectal cancer screening.

Marker	Performance				Studied in
	Stool		Blood		
	Sensitivity (%)	Specificity (%)	Sensitivity (%)	Specificity (%)	
miR-18a-5p	54.1	81.6	73.1	79.1	[32,33]
miR-19a-3p	53.3	89.1	-	-	[33]
miR-20a-5p	36	86.8	-	-	[32,33,34,35]
miR-21-5p	71.7	71.5	76.1	84.4	Nearly all papers
miR-27a-3p	69	52	58.9	88.2	[36,37,38]
miR-29a-3p	76.5	57	69	89.1	[24,38,39,40]
miR-92a-3p	64.8	72	74.8	81.5	[21,24,33,34]
miR-135b-5p	71.1	79	93.1	72.7	[33,41,42]
miR-223	68.3	83.7	-	-	[34,39,43,44]

Note: The reported sensitivities and specificities in Table 2 represent the reported mean (or sole) values from Santos et al. [16]. The final column indicates a selection of other publications that studied the miRNA in that row.

**Table 3:** A selection of published papers that report on miRNA ability to detect advanced adenomas, along with any reported sensitivity, specificity, and AUC.

Paper	miRNA(s)	Sensitivity for Advanced Adenomas (%)	Specificity for Advanced Adenomas (%)	AUC
Duran-Sanchon et al. (2020) [38]	miR-421	81	43	0.68
	miR-130b-3p	82	39	0.64
Huang et al. (2010) [23]	miR-29a and miR-92a	73	79.7	0.773
Imaoka et al. (2016) [62]	miR-1290	46.4	91.2	0.718
Zhao et al. (2022) [63]	miR-627-5p	62	93	0.84
	miR-199a-5p	90	53	0.76
Wu et al. (2014) [41]	miR-135b	73	68	0.71

Abbreviations: AUC: area under the curve (in ROC curve analysis).

## Data Availability

No new data were created or analyzed in this study. Data sharing is not applicable to this article.
